# Emulsified omega-3 fatty-acids modulate the symptoms of depressive disorder in children and adolescents: a pilot study

**DOI:** 10.1186/s13034-017-0167-2

**Published:** 2017-07-05

**Authors:** Jana Trebatická, Zuzana Hradečná, František Böhmer, Magdaléna Vaváková, Iveta Waczulíková, Iveta Garaiova, Ján Luha, Igor Škodáček, Ján Šuba, Zdeňka Ďuračková

**Affiliations:** 1 Department of Child and Adolescent Psychiatry, Faculty of Medicine, Comenius University and Child University Hospital, Limbová 1, 833 40 Bratislava, Slovakia; 2 Institute of Medical Chemistry, Biochemistry and Clinical Biochemistry, Faculty of Medicine, Comenius University, Sasinkova 2, 813 72 Bratislava, Slovakia; 3 Department of Nuclear Physics and Biophysics, Faculty of Mathematics, Physics and Informatics, Comenius University, Mlynská dolina F1, 842 48 Bratislava, Slovakia; 4 Research and Development Department, Cultech Ltd, Unit 2 Christchurch Road, Port Talbot, SA12 7BZ UK; 5 Institute of Medical Biology, Genetics and Clinical Genetics, Faculty of Medicine, Comenius University, Sasinkova 4, 813 72 Bratislava, Slovakia

## Abstract

**Background:**

The prevalence of mood disorders in children is a growing global concern. Omega-3 fatty acids (FA) are emerging as a promising adjuvant therapy for depressive disorder (DD) in paediatric patients. The primary objective of this pilot, single-centre, randomized, double-blind controlled study was to compare the efficacy of an Omega-3 FA fish oil emulsion with a control oil emulsion alongside standard treatment for depressive symptoms in children and adolescents suffering from depressive disorder (DD) and mixed anxiety depressive disorder (MADD).

**Methods:**

38 children (12 patients were treated and diagnosed for at least 1 month before enrolment, 26 patients were first-time diagnosed as DD) aged 11–17 years were randomised 1:1 to the intervention (Omega-3 FA, 19 patients) or active comparator (Omega-6 FA, 19 patients) groups. Children’s depression inventory (CDI) ratings were performed at baseline, every 2 weeks for a 12-week intervention period and at 4-week post-intervention. 35 patients (17 in Omega-3 and 18 in Omega-6 groups) who completed the whole intervention period were evaluated. Patients from Omega-3 group were stratified according to diagnosis into two subgroups (DD—10/17 and mixed anxiety depressive disorder (MADD)—7/17 patients) and in the Omega-6 group into DD—10/18 and MADD—8/18 patients. Groups were evaluated separately. Differences between-groups were tested with the Student´s *t* test or non-parametric Mann–Whitney U test. Two-way ANOVA with repeated measures and Friedman test were used to analyse the *Treatment* effect for response in CDI score. p < 0.05 was considered significant in all statistical analyses.

**Results:**

Significant reductions in CDI scores in 35 analysed patients who completed 12 weeks intervention were observed after 12 weeks of intervention only in the Omega-3 group (p = 0.034). After stratification to depressive disorder and mixed anxiety depressive disorder subgroups, the DD subgroup receiving the Omega-3 FA fish oil showed statistically greater improvement (score reduction after 8 week treatment of −9.1 CDI, p = 0.0001) when compared to the MADD subgroup (score reduction after 8 week treatment −4.24 CDI, p = 0.271).

**Conclusions:**

CDI scores were reduced in the Omega-3 group and the depression subgroup had greater improvement than the mixed depressive/anxiety group. An Omega-3 fatty acid rich fish oil emulsion may be an effective adjuvant supplement during the treatment of depressive disorders in children.

*Trial registration* ISRCTN81655012

**Electronic supplementary material:**

The online version of this article (doi:10.1186/s13034-017-0167-2) contains supplementary material, which is available to authorized users.

## Background

Mood disorders in children and adolescents are a serious global problem in child psychiatry and its incidence is shifting to younger years of age. The prevalence of depression is 5.7% among 13–18 years old with a female to male ratio of 1.3:1 [1].

Features of mood disorders in children include a pervasive and persistent sadness, irritability, decreased school performance, loss of interest and pleasure in social contacts, attention deficit, sleep problems, loss of appetite, abdominal pain, headache and suicidal tendency [2].

Optimal pharmacological management of child and adolescent depressive disorder should occur alongside educative and supportive psychotherapy. It is recommended that first line antidepressant medication, such as selective serotonin reuptake inhibitors (SSRI) including fluoxetine, sertraline, fluvoxamine and citalopram, are prescribed by a clinician with significant experience in the treatment of depression.

The molecular basis of depressive and anxiety disorders in children is not fully understood [3]. It is believed that the establishment and development of depressive disorder (DD) involves, among others, nutritional factors which contribute through the composition and content of lipids and lipid metabolism [4]. The increased incidence of DD in people of Western countries has been associated with drastic changes in dietary habits over the century in which the consumption of Omega-3 fatty acids (FA) in the form of fish, grain and vegetables has been replaced by the Omega-6 FA from cereal oils. The ratio of Omega-3 FA to Omega-6 FA in the diet has shifted from 1:1 to 1:15 and this switch has coincided with a strong rise in the rates of depression in recent decades [5]. This has led to the hypothesis that Omega-3 FA supplementation could represent an approach for treating depression and other mood disorders [6–8].

In recent years, research has been focused on the adjuvant therapy of depression with the aim of reducing the consumption of antidepressants, to prolong remission and improve prognosis in paediatric and adolescent patients [9]. A review by Clark et al. [10] concluded that adjuvant use of medication is sometimes appropriate in children and adolescents because tricyclic antidepressants are no benefit in adolescents and antidepressants have a boxed warning for the increased risk of suicide. The beneficial effects of adjuvant therapies, including some polyphenolic compounds [11] and Omega-3 fatty acids, have been demonstrated in the prevention and treatment of depression disorder [12–14], but not in mania [15].

During a meta-analysis of 10 double-blind and placebo controlled studies with a treatment period of 4 weeks or longer, Lin and Su [13] observed a significant antidepressant efficacy of Omega-3 FA in patients with clearly defined depression (ES = 0.69, p = 0.002) or with bipolar disorder (ES = 69, p = 0.0009). However, significant heterogeneity among these studies and publication bias were noted. For this reason more large-scale and well-controlled trials are recommended by authors to find out the favourable target subjects and appropriate therapeutic doses of Omega-3 FA [16, 17]. Omega-3 FA have been found to have no “mood-improving” effects [18] or antidepressant effect in patients with defined depressive disorder but not in patients with depression without diagnosis of DD [19, 20] and no evidence of positive effects were found in healthy subjects, patients with schizophrenia [21] or patients with Alzheimer disease [22]. In a meta-analysis of 13 randomized, placebo-controlled trials [17] examining the efficacy of Omega-3 FA involving 731 participants, no significant benefit of Omega-3 FA treatment compared to placebo were demonstrated although the analysed trials demonstrated significant heterogeneity and publication bias. The authors of this meta-analysis excluded Omega-3 FA trials of primary psychiatric disorder other than major depression (e.g. bipolar disorder, schizophrenia and obsessive–compulsive disorder) where depression was typically a secondary outcome and the reporting of secondary outcomes may be particularly prone to publication bias and a source for increased heterogeneity. Patients prior to enrolment in trials with Omega-3 FA should be accurately diagnosed according to ICD-10/DSM-IV with DD [20].

Conversely, a meta-analysis of depressive adult patients showed that levels of Omega-3 FA, eicosapentaenoic acid (C20:5, EPA) and docosahexaenoic acid (C22:6, DHA), are lower in these patients and indicate that this may play a role in the pathogenesis of depression [23].

Another meta-analysis of 11 trials conducted in adult individuals with diagnoses of major depression [24–26] and bipolar disorder [27, 28] provided further evidence that Omega-3 FA supplementation in combination with standard antidepressant therapy has beneficial clinical effects on depressive status. Results from this meta-analysis indicate that final clinical efficacy is influenced mainly by EPA, rather than DHA present in supplements [18, 29]. In a dose-ranging study involving adult patients with persistent depression supplemented with ethyl-eicosapentaenoate as an adjuvant therapy, Peet and Horrobin [30] found that the efficacy was dose dependent and a dosage of 1 g/day was effective in treating depression. Martins et al. [31] also demonstrated a dose–response relationship for EPA efficacy in meta-regression analysis and concluded that if EPA is to be further evaluated as an antidepressant, then doses of up to 4.4 g/day should be used in adults. However, another meta-analysis of depressive adults suggests a small-to-modest non-clinically beneficial effect of Omega-3 fatty acids on depressive symptomatology compared to placebo [32].

It is proposed that the effect of Omega-3 FA is based on modulating membrane fluidity and their anti-inflammatory effects through formation of anti-inflammatory eicosanoids [8] and/or protective resolvins and docosanoids [33]. Recent meta-analysis [34] suggests a promotional effect of Omega-3 FA on the effect of antidepressants through modulation of neuronal membrane-antidepressant interactions or influencing the antidepressant transport across the blood–brain barrier by influencing p-glycoprotein. However, according to Clark et al. [10] the extrapolation of adult data on antidepressant medication to children and adolescent may not be accurate, because neural pathways may not be fully developed and serotonin and norepinephrine system have different maturation rates [35]. Currently, there is a lack of information regarding the impact of Omega-3 fatty acids on depression symptoms in children although two pilot studies have examined the impact of Omega-3 FA supplementation in paediatric patients with depressive disorders [36]. Nemets et al. [37] investigated children (aged 6–12 years) with major depressive disorder and daily supplementation with either one 1000 mg capsule containing EPA (400 mg) and DHA (200 mg) or two 500 mg capsules containing EPA (190 mg) and DHA (90 mg), depending on their ability to swallow a larger capsule, for a period of 16 weeks with a safflower oil/olive oil placebo. The 20 patients who completed at least 1 month intervention showed, in contrast to the placebo, significant improvements in children’s depression rating scale (CDRS) at weeks 8, 12 and 16 of intervention (least significant difference post hoc test, p = 0.04, 0.03 and 0.03, resp.). CDI and CGI (clinical global impression) scores were similar to CDRS. Furthermore, McNamara et al. [38] performed a 10 weeks open label trial with 8–24 years old adolescents with SSRI treatment-resistant major depressive disorder and observed a significant (40%) reduction (p < 0.001) in symptoms in those receiving a high dose of fish oil (16.2 g/day; 10.8 g EPA, 5.4 g DHA) whilst those receiving a lower dose (2.4 g/day; 1.6 g EPA, 0.8 g DHA) showed a trend towards symptom reduction (20%) (p = 0.06).

The effects of Omega-3 FA has also been investigated in 18 children and adolescents with juvenile bipolar disorder. After 6 weeks of daily EPA (360 mg) and DHA (1560 mg) supplementation in an open-label study, the clinical ratings of mania and depression were significantly lower [39]. In another recent randomized, double-blind, controlled trial the combined treatment of Omega-3 FA plus inositol reduced symptoms of mania and depression in 10 pre-pubertal children with mild to moderate bipolar spectrum disorders [40].

In the study by Amminger et al. [41], Omega-3 FA significantly reduced both the positive and negative symptoms and improved functioning in adolescents with high risk of psychosis compared with placebo, but no significant effect was observed on depressive symptoms.

The two mentioned works [37, 38] are pilot studies and it is therefore difficult to compare them with our project for differences between studies (forms of supplement—capsules versus oil emulsion, doses, the content of EPA and DHA and the duration of the intervention).

There is also insufficient data on gender sensitivity to supplementation with Omega-3 fatty acids in depressed children. In the study of Murakami et al. [42] it is stated that fish intake in boys was inversely associated with depressive symptoms (p = 0.04) and that EPA intake, but not DHA intake, is negatively associated with depressive symptoms (p = 0.04 for EPA and p = 0.11 for DHA). Conversely, no such associations were observed among girls.

The primary objective of this pilot, single-centre, randomized, double-blind, and active-controlled study was to compare the efficacy of Omega-3 FA with Omega-6 FA present as oil emulsions in the treatment of depressive symptoms in children and adolescents suffering from depressive disorder and mixed anxiety depressive disorder.

## Methods

### Subjects

Thirty eight out-patients (8 boys and 30 girls) suffering from depressive disorder (n = 21) or mixed anxiety and depressive disorder (MADD) (n = 17) registered at the Department of Child and Adolescent Psychiatry of the Faculty of Medicine of Comenius University and the Child University Hospital between June 2013–December 2015, were enrolled in this prospective study.

Inclusion criteria included diagnosis of depressive disorder or mixed anxiety and depressive disorder, age 7–18 years, with no indication of chronic somatic disease and normal eating habits. The diagnoses were determined according to International Classification of Diseases, 10th edition (ICD 10).

Exclusion criteria were chronic somatic diseases (endocrine, metabolic, autoimmune), dietary restrictions (vegetarians, lactose intolerance, celiac disease), psychotic disorders, eating disorders, addiction to psychoactive compounds, personality disorders, organic mental disorders and pervasive developmental disorders.

All out-patients, and parents of, who were managed at the out-patients’ clinic of the University Hospital and met diagnostic criteria of depressive disorder were informed about possibility to take part in the current trial. 74 patients and their parents were addressed and 38 patients met the inclusion criteria and agreed to take part in the study (Additional file 1).

Written informed consent was obtained from parents or legal guardians prior to participation in the study. Children gave verbal assent prior to enrolment in the trial.

### Study design and intervention

Patients were randomized to receive either an Oomega-3 fatty acids rich fish oil emulsion (Omega-3 FA) or an active comparator Omega-6 FA rich sunflower oil emulsion for 12 weeks followed by a wash-out period (4 weeks). Children were included in the study according to ICD 10 with the following diagnoses: depressive disorder (DD, n = 21; 61.8%) and mixed anxiety and depressive disorder (MADD, n = 17; 38.2%).

Alongside their standard antidepressant therapy, children received daily either 20 mL of Omega-3 fish oil emulsion (providing 2400 mg of total Omega-3 FA; 1000 mg EPA and 750 mg DHA, EPA:DHA ratio = 1.33:1) or an identically looking comparator Omega-6 sunflower oil emulsion containing 2467 mg of Omega-6 linoleic acid provided by Cultech Ltd, Port Talbot, UK. The dose of Omega-3 FA used was determined based on a review of the literature. Compliance to product was assessed by monitoring volume of intervention returned and was above 95%.

### Randomisation

Trial participants were allocated in a 1:1 ratio to the two arms (Omega-3 and Omega-6) according to a computer-generated random sequence using block randomisation with a block-size of four. The randomisation was performed by an independent statistician. Patients were enrolled and assigned sequentially to supplement interventions by the physician. The allocation sequence was not available to any member of the research team until the databases had been completed and locked.

### Data collection

Patients characteristics (age, gender, menstruation in female) and relevant clinical variables (treatment history—duration of disease/firstly diagnosed, treatment/no treatment, current medication/no medication with antidepressants) were recorded for each patient.

Clinical examinations of all participants were implemented as follows: at the beginning of the trial (week 0) and every 2 weeks for 3 months (weeks 2, 4, 6, 8, 10, 12). The last examination was performed at the week 16, after the 4 week wash-out period. The process of data collection is graphically depicted in a Consort flow diagram (Additional file 1).

Only data from patients who completed 12-weeks of intervention were analysed. Patients who discontinued the study before the week 12 were excluded from evaluation.

Ratings were made using the self-rated scale Children’s Depression Inventory (CDI) [43, 44] with a higher CDI score representing a higher depressive state.

### Anthropometric assessment

Body weight and height were measured without shoes and with light clothing using a digital weighing and measuring station with automatic body mass index (BMI) calculation (kg/m^2^, SECA 764, Germany).

### Data management and analysis

#### Sample size estimation

As a pilot study there was no formal sample size calculation.

#### Statistical analysis

Descriptive and univariate analyses were performed on all selected patients’ characteristics. Mean ± standard deviation (SD) is given for the normally distributed variables or a median and interquartile range for data showing departures from normality. Categorical variables are presented as counts and percentages.

First, the treatment groups were tested for between-group differences in all relevant baseline characteristics (age, gender, type of diagnosis, and CDI score). Symmetrical data were analysed with the Student´s *t* test for independent samples. If the data were skewed but other criteria were met, a non-parametric Mann–Whitney U test was used. Due to differences in the outcome of testing two sample Smirnov test for distribution differences was performed.

Two-way ANOVA with repeated measures was used to analyse *Treatment* effect (main factor effect) in patients who were repeatedly evaluated for response in CDI scores (*Time* was a factor). CDI scores were obtained at the beginning of the study (the baseline) and then biweekly for the rest of the study (up to 12 weeks).

Significant interaction between treatment and time was taken as an evidence of difference between CDI outcomes under the two treatment conditions (Omega-3 and Omega-6 FA). Differences between the baseline values of each patient and the following time points were then assessed with the Friedman test.

A value p < 0.05 was considered significant in all statistical analyses. For statistical analysis we employed the statistical programs StatsDirect^®^ 2.8.0 (StatsDirect Sales, Sale, Cheshire, M33 3UY, UK) and IBM SPSS Statistics 23. Graphical representation of data was made using program Excel 2010 (Microsoft Co.).

## Results

### Enrolment and baseline characteristics

Patient characteristics such as age (15.5 ± 1.5 years, 11–17 years), gender, and relevant clinical variables (e.g. treatment history and current medications) were summarised in order to characterise the study population and to judge baseline comparability of the treatment groups (Additional file 1).

From the 38 patients included in our study, three dropped out at an early stage (after 1–2 days after the enrolment) due to product palatability (2 patients from the Omega-3 group) and for non-compliance (reluctance to miss school every 2 weeks in order to visit the clinic, difficulties with transportation of outside city patients; one from the Omega-6 group).

The 35 patients (average age 15.5 years) who completed intervention were included in data analysis (17 in Omega-3, 12 F and 5 M and 18 in Omega-6, 15 F and 3 M).

In order to determine whether omega-3 shows an improvement in the depressive symptoms rating as CDI score, we analyzed the data of the patients who fully underwent the intervention. In parallel, all randomized subjects were analyzed according to randomization. Dropped out patients (n = 3, two from the omega-3 group and one from the omega-6 group) were analyzed using their baseline values (patients dropped out after 1–2 days after the study enrollment). No statistical significant difference was observed between analyses and results have led to the same conclusion.

Isolated missing data (n = 3, 2.2% in omega-3 group and n = 2, 0.14% in omega-6 group) was replaced with the average of the values obtained from the previous and the following week.

Three patients from the Omega-6 group dropped out after 12 weeks (after termination of fatty acids supplementation). 20 out of 35 patients from both groups were diagnosed as DD (57.1%) and 15 out of 35 patients as MADD (42.9%). One patient from Omega-3 was diagnosed as depressive disorder with social phobia as comorbidity. From the evaluated patients (n = 35), 12 patients (7 from Omega-3 and 5 from Omega-6 FA groups) were diagnosed and treated for at least 1 month before the intervention with SSRI antidepressant pharmacotherapy (sertraline, fluvoxamine, fluoxetine) and 23 patients were first-time diagnosed with depressive disorder (10 were from Omega-3 and 13 from Omega-6 groups). Of these, 10 children were not treated with SSRIs during the intervention (4 patients from Omega-3 group and 6 from Omega-6 group). The remaining 25 children (13 patients from Omega-3 group and 12 from Omega-6 group) were medicated during intervention with SSRIs and FA simultaneously (Additional file 1). Patients used sertraline in the dose range 50–150 mg, fluvoxamine at the dose of 50 mg, fluoxetine at the dose range 20–60 mg and carbamazepine at the dose of 150 mg per day (1 patient). All patients were given the supportive therapy and psychoeducation.

### Basic anthropometric

Data were evaluated in patients with depression (Table 1).

**Table 1:** Anthropometric parameters of depressive patients

Parameter	Evaluated patients				Omega-3 group			Omega-6 group		
	All	Male	Female	p M/F	Male	Female	p M/F	Male	Female	p M/F
Number (n)	35	8	27		5	12		3	15	
Age (years)	15.6 ± 1.6	16.4 ± 2.2	15.3 ± 1.3	n.s.	16.3 ± 1.5	15.4 ± 1.4	n.s.	16.5 ± 1.6	15.3 ± 1.4	n.s.
Weight (kg)	59.6 ± 11.9	68.2 ± 15.5	56.9 ± 9.4	0.015	68.3 ± 15.5	56.7 ± 9.4	0.04	67.8 ± 15.3	57.2 ± 9.1	0.03
Height (m)	1.68 ± 0.1	1.74 ± 01	1.66 ± 0.1	0.016	1.75 ± 0.1	1.65 ± 0.1	0.02	1.73 ± 0.1	1.67 ± 0.1	0.02
BMI (kg/m^2^)	21.1 ± 3.1	22.4 ± 3.6	20.6 ± 2.9	n.s.	22.05 ± 3.1	20.8 ± 2.8	n.s.	22.7 ± 3.7	20.15 ± 2.5	n.s.

*M* male, *F* female, *p* significance, *n.s.* nonsignificant

### Clinical parameters

#### Baseline

All individuals’ characteristics were examined for between-group differences at the start of the study. The groups were not statistically different with respect to age (p = 0.958), gender composition (p = 0.69) and type of diagnosis (DD and MADD), p = 0.999).

No gender differences in CDI score were observed at the baseline (p = 0.208). No significant differences were found in the mean age between girls (mean 15.3 ± 1.3 year) and boys (mean 16.4 ± 2.2 year) nor in the mean baseline CDI scores for girls and boys, 24.95 ± 10.07 and 20.25 ± 8.46, respectively. Due to differences in the outcome of testing, we performed two sample Smirnov test for distribution differences and did not identify any serious shift in the CDI value distribution between girls and boys (p = 0.217). The proportion of cases with depressive disorder (57.1% of total 35 evaluated patients) at baseline was non-significantly (p = 0.31) higher than the proportion of cases with depression plus anxiety (MADD) (42.9%). At baseline there were no significant difference in CDI scores between patients with a first depressive episode (23 out of 35 patients, 10 in the Omega-3 and 13 in the Omega-6 groups) and already treated patients (12 out of 35 patients, 7 in the Omega-3 and 5 in the Omega-6 groups, p = 0.124, see Consort flow diagram, Additional file 1). The mean baseline CDI score for the Omega-3 group was 26.8 ± 8.1 (median 27 points with Q1–Q3 of 17–34) and 21.2 ± 7.4 for the Omega-6 group (median 19 points with Q1–Q3 of 14–28). Both mean score values were in the range of moderate and severe depression severity [44, 45]. Randomisation was strictly blinded and higher CDI score at baseline in Omega-3 group compared to the Omega-6 group was not significant (p = 0.94).

#### The impact of intervention

The impact of Omega-3/Omega-6 FA on the CDI score of patients after 6 and 12 weeks of intervention and after the wash-out period (time 16) is shown in Additional file 2: Table S2.

**Fig. 1:**
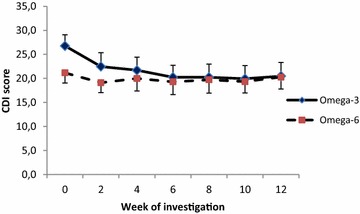
Treatment effect on CDI score (±SE) in the Omega-3 and Omega-6 groups. n (Omega-3 group) = 17, n (Omega-6 group) = 18, *SE* standard error

Significant differences (p = 0.001) were seen between the two treatment groups (Omega-3 and Omega-6) with regards to *Time.* Significant differences were also detected between Omega-3 and Omega- 6 groups for the interaction of Treatment*Time (p = 0.034). Figure 1 shows the CDI scores in 35 patients who completed 12 weeks of intervention period.

**Fig. 2:**
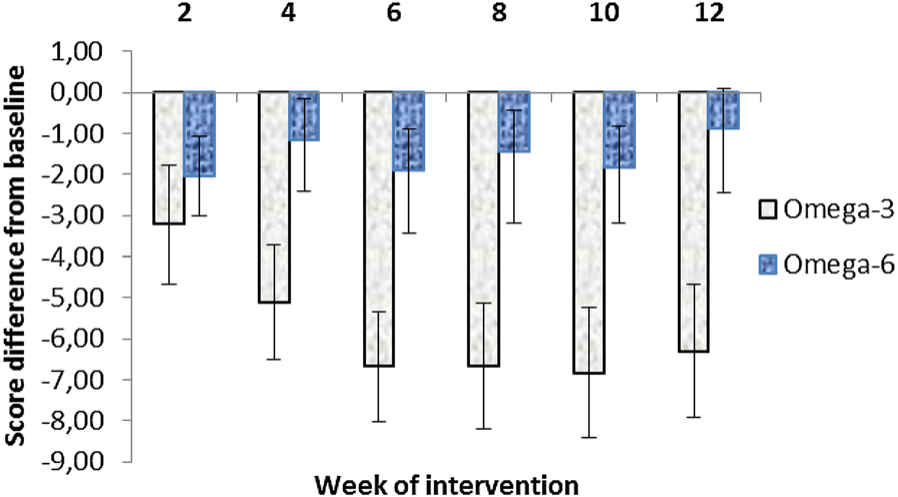
Pairwise comparison of fatty acid effects as the difference between CDI score (±SE) at the baseline and the week of investigation. n (Omega-3 group) = 17, n (Omega-6 group) = 18

The effect of time-dependent treatment in the Omega-3 group as determined by the Friedman test is also significant (F = 3.87, df = 6, p = 0.0017) in the contrast to Omega-6 group (F = 0.36, df = 6, p = 0.904). All pairwise comparisons (Conover) in the Omega-3 group showed highly significant differences from baseline (from p = 0.005 in the 2nd week to p = 0.0001 at week 12). The highest reduction of CDI score in the Omega-3 group was observed after 10 weeks of intervention (−6.82) representing a −25.5% of baseline score (Additional file 2: Table S2; Fig. 2).

In the Omega-6 group no significant effect of either the treatment or the pairwise comparison was observed. The highest reduction of CDI score in the Omega-6 group was observed after 6 weeks of intervention (−1.89), representing a −8.9% of baseline score (Additional file 2: Table S2; Fig. 2).

**Fig. 3:**
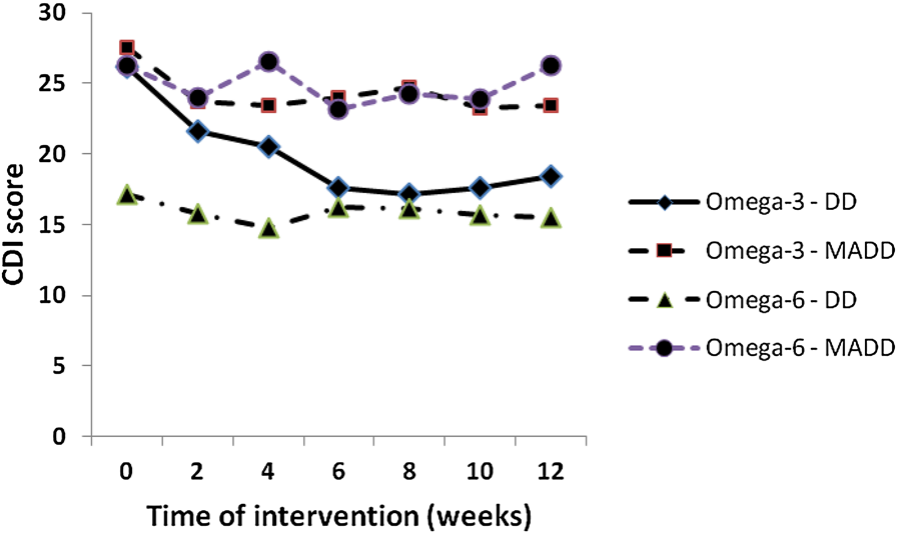
Treatment effect on CDI score in the Omega-3 and Omega-6 groups in patients with DD and MADD diagnoses. *DD* depressive disorder (n = 10), *MADD* mixed anxiety and depressive disorder (n = 7)

Since both groups comprised patients of two clinically different conditions, depressive disorder (DD) or mixed anxiety and depression disorder (MADD), we evaluated the difference in treatment response between DD and MADD subgroups. Significant differences (p = 0.032) were seen between the two diagnoses in the Omega-3 groups with regards to *Time.* The interaction between Treatment*Time in the Omega-3 group for two different diagnoses showed a trend towards significance for only the DD subgroup (p = 0.095). Due to the small number of patients in the diagnose subgroups (10 of 17 patients with DD and 7 of 17 with MADD) we examined the difference in CDI score over time using the Friedman post hoc test. The reduction of CDI score in the Omega-3 group was higher in subgroup of patients with DD diagnosis (−9.1 in CDI equating to a 34.7% reduction from baseline in 8th week of intervention, n = 10, F = 5.3, df = 6, p = 0.0001) in comparison to MADD diagnosis (−4.24 in CDI score equating to 15.5% reduction from baseline after 10 weeks of intervention, n = 7, F = 0.59, df = 6, p = 0.271) (Additional file 3: Table S3; Fig. 3).

The effect of treatment determined by two-way ANOVA with repeated measures in both groups (Omega-3 and Omega-6) was not dependent on age (p = 0.232), gender (p = 0.163) or newly diagnosed or patients already treated with antidepressants (p = 0.205).

The change of CDI score from weeks 12 to 16 (1 month after termination of oil emulsion administration) was not significant in both the Omega-3 and Omega-6 groups.

## Discussion

The present study investigates the effect of treatment with an Omega-3 fatty acid fish oil emulsion containing EPA and DHA compared with an Omega-6 FA sunflower oil emulsion on depressive disorder in children and adolescents and we found significant reductions in CDI scores in 35 analysed patients who completed 12 weeks intervention with the Omega-3 FA emulsion (p = 0.034). After stratification to subgroups with depressive disorder and mixed anxiety depressive disorder, the DD subgroup receiving the Omega-3 FA showed statistically significant greater improvement (p = 0.0001) when compared to the MADD subgroup (p = 0.271).

Both emulsions were well tolerated and no serious adverse side effects were recorded. Only one patient from the Omega-3 group stated more frequent defecation (2–3 × daily).

Three subjects discontinued from the study before week 6 (two from the Omega-6 and one from the group Omega-3 groups) for non-compliance (timidity of blood collection, reluctance to miss school during examinations, difficulties with transportation of outside city patients) and the reasons were recorded by the child psychiatrist in medical records.

The Omega-3 FA emulsion used in our trial contained 57.2% EPA and 42.8% DHA in line with the recommendations of Hallahan et al. [20], Lin et al. [18] or Sublette et al. [29], who recommended higher doses of EPA than DHA (˃50 or ˃60% EPA, respectively of total EPA + DHA). The reason for higher amount of EPA is not quite known, but it is assumed that anti-inflammation and anti-oxidative effects are involved in the protection mechanisms [8, 46–48]. The daily dose of Omega-3 FA in our study was higher (2400 mg fish oil present in an emulsified form) compared to the dose used by Nemets et al. [37] that used 1000 mg of encapsulated fish oil. Our dose was comparable with lower dose of 2.4 g/day used by McNamara et al. [38] although FA from emulsions can be absorbed more effectively compared to capsules [49, 50]. In our study, all patients, except one female patient in the Omega-6 group, were in pubertal age (menstruation). The male to female ratio was 5 M/12 F in the Omega-3 group and 3 M/15 F in the Omega-6 group. In the study by Nemets et al. [37]) children were of preadolescent age with male to female ratio of 7:3. We did not find any gender and age dependence in the Omega-3 FA effect. Similarly, Kovacs et al. [43] concluded that studies examining gender and age differences on CDI symptoms were inconsistent showing no clear age or gender effects. In the study by Masip et al. [51], girls scored higher than boys on CDI symptoms. Adolescent girls had higher rates of depression than adolescent boys whereas preadolescent boys and girls reported similar levels of depressive symptoms. Thus, the age of the participants may have been a factor in attenuating the gender differences [52].

In a 16 week, randomized, double-blind, placebo-controlled study, Nemets et al. [37] found a significant difference from the placebo (sunflower oil or olive oil) in the CDI score, CDRS and clinical global impression after 8 weeks of intervention with a fish oil capsule (1000 mg containing 400 mg EPA and 200 mg DHA or two 500 mg capsules with 190 mg EPA and 90 mg DHA) in 20 children diagnosed with major depression (5/10 drop-out in placebo and 3/10 in Omega-3 group). Seven out of 10 patients from the Omega-3 group experienced a greater than 50% reduction in CDRS, compared to none in placebo group with four meeting the criteria for remission.

In our study the greatest effect occurred between weeks 6 and 12 of the intervention period (from 24.4 to 25.5% reduction of CDI score) in the Omega-3 group in contrast to the Omega-6 group where the highest reduction of CDI score (8.9%) was observed after 6 weeks of intervention. Although the randomisation was strictly blinded and psychiatrists classify patients according to the rising score and do not recognize the affiliation with the Omega-3/Omega-6 groups, a non-significantly increase in CDI score was recognized in the Omega-3 group (26.8 ± 8.1 versus 21.2 ± 7.4, p = 0.094). However, results published in a recent meta-analysis by Mocking et al. [34] concluded that the Omega-3 FA response was independent of baseline depressive severity but it is worthy of note that this meta-analysis evaluated results from adults and its relevance to children is still unknown.

Results from our work and also the work by Nemets et al. [37] suggest a significant improvement of the CDI score after the intervention with Omega-3 fatty acids. We also detected differences in sensitivity to the Omega-3 fish oil emulsion between subgroups of patients with depressive disorder and mixed anxiety and depressive disorder. After stratification to DD and MADD subgroups, the DD subgroup had a 34.7% decrease in CDI score from baseline (p < 0.0002) in the contrast to MADD subgroup of the Omega-3 group where 15.5% reduction of CDI score was observed after 10 weeks (p = 0.732).

Similarly to our results, Lespérance et al. [53] observed different efficacy of Omega-3 FA (1050 mg of EPA and 150 mg of DHA/day) on major depression and major depression with comorbid anxiety where the latter was less sensitive to Omega-3 FA treatment. However, the relatively contradictory results of Liu et al. [54] confirmed association between the presence and severity of comorbid anxiety with the lowest EPA and DHA levels while depressive severity was not associated with plasma polyunsaturated fatty acid levels.

In our study, a limited number of patients without standard antidepressant treatment during intervention were included (FA as a monotherapy, 4 patients of 17 in the Omega-3 group and 6 patients of 18 in the Omega-6 group). The positive effect of Omega-3 FA in children in our study represents an adjuvant effect to standard antidepressant therapy and is in agreement with published findings of Grosso et al. [19] and Mocking et al. [34] that compared the efficacy of Omega-3 FA as a mono-therapy versus adjuvant therapy in adults. Both meta-analyses found significant effects when the Omega-3 FA was administered together with the standard antidepressant therapy.

The effect of Omega-3 FA on depressive symptoms of children and adolescents (8–24 years old) was also investigated by McNamara et al. [38] in an open-label study. The authors evaluated depressive symptoms with the CDRS-R scale in 7 patients supplemented with a daily low dose of fish oil (2.4 g of Omega-3 FA containing 1.6 g EPA and 0.8 g DHA) or with a daily high dose of fish oil (16.2 g/day of Omega-3 FA containing 10.8 g EPA and 5.4 g DHA). Significant improvement was detected only in the higher dose group (the score decreased by 40%) whereas supplementation with the lower dose showed a trend of improvement (by 20%). The dosages of Omega-3 FA used in our study are markedly lower than the higher dose of McNamara´s work and our study did not include SSRI resistant patients.

The current study, however, suffers from several limitations:

- The selection of an appropriate reference treatment (a suitable control) is problematic as other palatable oils such as olive oil and sunflower oils are known to exhibit different bio-modulating activities. Olive oil contents many different antioxidants and biomodulating polyphenolic compounds which can influence different metabolic processes. Sunflower oil with its polyunsaturated fatty acids (linoleic acid) can modulate (similarly than oils with omega-3 FA) for example fluidity of membrane and by this way to influence transport of neurotransmitter and other compounds through membrane. For these reasons, an ideal active comparator for omega-3 FA is not available.
- The small sample size and use of a single clinical setting for recruitment of patients.
- Ratings were made using only one type of scale—children’s depression inventory (CDI). Although minimally three type of rating scales are available and used for children in some countries (children’s depression rating scale, CDRS, clinical global impression, CGI and children’s depression inventory, CDI), only one type of scale for rating of depressive symptoms (CDI) is validated for children and adolescents in Slovakia.
- An imbalance between male (n = 8) and female (n = 27) patients.

## Conclusions

Results from our study show that Omega-3 fatty acid rich fish oil may be an effective adjuvant supplement to standard antidepressant therapy for the treatment of depressive disorder, rather than mixed anxiety and depressive disorder, in children and adolescents and corroborates observations made by numerous other studies in adult cohorts. Due to the small number of participants it is difficult to draw solid conclusions and recommendations from these results for paediatric clinical praxis for efficacy of Omega-3 fatty acids on depressive symptoms in children although clear beneficial effects were observed. This study also highlights the necessity for larger randomised studies in order to translate these preliminary findings into the clinical setting.

## Additional files

**Additional file 1.** Consort flow diagram.

**Additional file 2: Table S2.** CDI score in the Omega-3 and Omega-6 groups at different weeks of Intervention. SD – standard deviation, n – number of subjects, a – p value between the week 12 and 16.

**Additional file 3: Table S3.** CDI score in the Omega-3 and Omega-6 groups at different diagnosis and weeks of intervention. DD – depressive disorder, MADD – mixed anxiety and depressive disorder, a – p value between week 12 and 16, SD – standard deviation, n – number of subjects.

## Abbreviations

CDI: children’s depression inventory
CDRS: children’s depression rating scale
DD: depressive disorder
DHA: docosahexaenoic acid
EPA: eicosapentaenoic acid
F: female
FA: fatty acids
ICD: international classification of diseases
M: male
MADD: mixed anxiety depression disorder
P: level of statistical significance
SD: standard deviation
SE: standard error
SSRI: selective serotonin reuptake inhibitor
