# Supplementary material for: Emulsified omega-3 fatty-acids modulate the symptoms of depressive disorder in children and adolescents: a pilot study
Source: Child Adolesc Psychiatry Ment Health. 2017 Jul 5;11:30. doi: 10.1186/s13034-017-0167-2 (PMC5497377; doi:10.1186/s13034-017-0167-2)
Supplement: Supplementary file 1 — Additional file 1. Consort flow diagram. [file 13034_2017_167_MOESM1_ESM.doc]

**
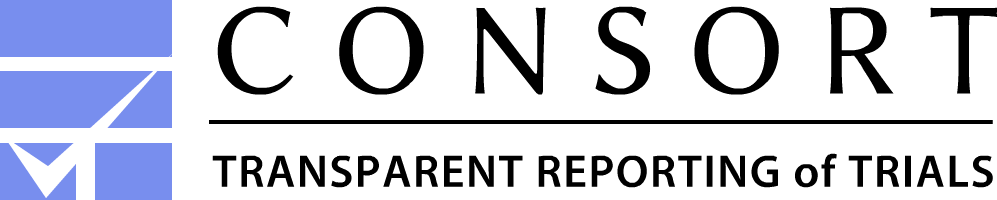
**

**CONSORT 2010 Flow Diagram**

**Enrollment**

**Allocation**

**Investigation**

**Follow-Up**

Excluded (n=36)

♦  Not meeting inclusion criteria (n=11)

♦  Declined to participate (n=10)

♦  Travelling problems (n=6)

♦  Intolerance to blood taking (n=9)

Analysed (n=17)
♦ Excluded from analysis (n=0)

- Treated with SSRI (n=13)
- No treatment with SSRI (n=4)

Randomized (n=38)

Analysed (n=18)
♦ Excluded from analysis (n=0)

- Treated with SSRI (n=12)
- No treatment with SSRI (n=6)

**Omega 6** (n= 19)

♦ Received allocated intervention (n=18)

♦ Did not receive intervention whole period (n=1)

          Non-compliance (n=1)

- Subgroup with DD (n=10)
- Subgroup with MADD (n=8)
- Treated for at least one month before enrollment (n=5)
- Firstly diagnosed (n=13)

**Discontinued participation after week 12** (n=3)

- Non-compliance (n=1)
- Problem with travelling (n=1)
- Intolerance to blood taking (n=1)

**Omega 3** (n= 19)

♦ Received allocated intervention (n= 17)

♦ Did not receive intervention whole period (n=2)

Taste of supplement (n=2)

- Subgroup with DD (n=10)
- Subgroup with MADD (n=7)
- Treated for at least one month before enrollment (n=7)
- Firstly diagnosed (n=10)

**Discontinued participation after week 12** (n=1)

- Non-compliance (n=1)

Assessed for eligibility (n=74)
