# Supplementary material for: Emulsified omega-3 fatty-acids modulate the symptoms of depressive disorder in children and adolescents: a pilot study
Source: Child Adolesc Psychiatry Ment Health. 2017 Jul 5;11:30. doi: 10.1186/s13034-017-0167-2 (PMC5497377; doi:10.1186/s13034-017-0167-2)
Supplement: Supplementary file 2 — Additional file 2: Table S2. CDI score in the Omega-3 and Omega-6 groups at different weeks of Intervention. SD – standard deviation, n – number of subjects, a – p value between the week 12 and 16. [file 13034_2017_167_MOESM2_ESM.docx]

| Table S2 |  |  |  |  |  |  |  |  |  |
| --- | --- | --- | --- | --- | --- | --- | --- | --- | --- |
|  |  | Time of intervention (weeks) | | | | | | | Wash-out |
| **Intervention** |  | **0** | **2** | **4** | **6** | **8** | **10** | **12** | **16** |
| **Omega-3** | Average CDI | 26.8 | 22.5 | 21.7 | 20.2 | 20.2 | 19.9 | 20.5 | 21 |
|  | SD | 9.59 | 11.88 | 11.20 | 10.29 | 11.28 | 11.26 | 11.83 | 15.80 |
|  | n | 17 | 17 | 17 | 17 | 17 | 17 | 17 | 15 |
|  | p week vs 0 |  | **0.005** | **0.001** | **0.000** | **0.001** | **0.001** | **0.001** | 0.155^a^ |
|  | % of baseline |  | -16.0 | -18.9 | -24.4 | -24.4 | -25.5 | -23.5 |  |
| **Omega-6** | Average CDI | 21.2 | 19.1 | 20.0 | 19.3 | 19.7 | 19.3 | 20.3 | 19.1 |
|  | SD | 8.94 | 8.76 | 11.05 | 11.18 | 11.90 | 9.93 | 10.54 | 10.02 |
|  | n | 18 | 18 | 18 | 18 | 18 | 18 | 18 | 15 |
|  | p week vs 0 |  | **0.05** | **0.355** | **0.502** | **0.453** | **0.690** | **0.226** | 0.723^a^ |
|  | % of baseline |  | -9.71 | -5.51 | -8.92 | -6.82 | -8.66 | -4.20 |  |
| a - p value between week 12 and 16 | | |  |  |  |  |  |  |  |
