# Supplementary material for: Emulsified omega-3 fatty-acids modulate the symptoms of depressive disorder in children and adolescents: a pilot study
Source: Child Adolesc Psychiatry Ment Health. 2017 Jul 5;11:30. doi: 10.1186/s13034-017-0167-2 (PMC5497377; doi:10.1186/s13034-017-0167-2)
Supplement: Supplementary file 3 — Additional file 3: Table S3. CDI score in the Omega-3 and Omega-6 groups at different diagnosis and weeks of intervention. DD – depressive disorder, MADD – mixed anxiety and depressive disorder, a – p value between week 12 and 16, SD – standard deviation, n – number of subjects. [file 13034_2017_167_MOESM3_ESM.docx]

Table S3

| **Intervention with** |  | Time of intervention (weeks) | | | | | | | | | Wash-out |
| --- | --- | --- | --- | --- | --- | --- | --- | --- | --- | --- | --- |
| **Omega-3** |  | **0** | **2** | | **4** | | **6** | **8** | **10** | **12** | **16** |
| **Diagnose DD** | Average CDI | 26.20 | 21.60 | | 20.50 | | 17.60 | 17.10 | 17.60 | 18.40 | 21.0 |
|  | SD | 9.0 | 12.1 | | 11.4 | | 8.5 | 9.6 | 10.6 | 13.1 | 13,8 |
|  | n | 10 | 10 | | 10 | | 10 | 10 | 10 | 10 | 9 |
|  | p week vs 0 |  | **0.049** | | **0.023** | | **0.001** | **0.000** | **0.000** | **0.002** | 0.398^a^ |
|  | % of baseline |  | -17.6 | | -21.8 | | -32.8 | -34.7 | -32.8 | -29.8 |  |
| **Diagnose MADD** | Average CDI | 27.57 | 23.71 | | 23.43 | | 24.00 | 24.71 | 23.29 | 23.43 | 27.43 |
|  | SD | 10.6 | 12.1 | | 11.3 | | 11.9 | 12.0 | 11.4 | 9.0 | 17.4 |
|  | n | 7 | 7 | | 7 | | 7 | 7 | 7 | 7 | 7 |
|  | p week vs 0 |  | **0.125** | | **0.060** | | **0.098** | **0.263** | **0.271** | **0.222** | 0.383^a^ |
|  | % of baseline |  | -13.99 | | -15.03 | | -12.95 | -10.36 | -15.54 | -15.03 |  |
| **Omega-6** |  | **0** | **2** | | **4** | | **6** | **8** | **10** | **12** | **16** |
| **Diagnose DD** | Average CDI | 17.10 | 15.80 | | 14.80 | | 16.20 | 16.10 | 15.70 | 15.50 | 17.4 |
|  | SD | 7.7 | 7.5 | | 8.5 | | 11.4 | 11.5 | 10.2 | 10.8 | 10.8 |
|  | n | 10 | 10 | | 10 | | 10 | 10 | 10 | 10 | 9 |
|  | p week vs 0 |  | **0.619** | | **0.413** | | **0.262** | **0.346** | **0.322** | **0.383** | 0.676^a^ |
|  | % of baseline |  | -7.6 | | -13.5 | | -5.3 | -5.8 | -2.5 | -9.4 |  |
| **Diagnose MADD** | Average CDI | 26.25 | 23.25 | | 26.50 | | 23.13 | 24.25 | 23.88 | 26.25 | 21.50 |
|  | SD | 7.6 | 8.5 | | 10.5 | | 9.6 | 10.8 | 7.5 | 6.3 | 8.1 |
|  | n | 8 | 8 | | 8 | | 8 | 8 | 8 | 8 | 6 |
|  | p week vs 0 |  | **0.066** | | **0.884** | | **0.115** | **0.979** | **0.179** | **1.000** | 0.350^a^ |
|  | % of baseline |  | -11.43 | | 0.95 | | -11.90 | -7.62 | -9.05 | 0.00 |  |
|  | | | |  | |  |  |  |  |  |  |
